# Supplementary material for: An individualized protein-based prognostic model to stratify pediatric patients with papillary thyroid carcinoma
Source: Nat Commun. 2024 Apr 26;15:3560. doi: 10.1038/s41467-024-47926-w (PMC11053152; doi:10.1038/s41467-024-47926-w)
Supplement: Supplementary file 1 — Supplementary Information [file 41467_2024_47926_MOESM1_ESM.pdf]

Supplementary Information

An individualized protein-based prognostic model to stratify pediatric patients with papillary thyroid carcinoma

Zhihong Wang[1#], He Wang[2,3,4#], Yan Zhou[2,3,4#], Lu Li[2,3,4,5], Mengge Lyu[2,3,4], Chunlong Wu[6], Tianen He[2,3,4], Lingling Tan[6], Yi Zhu[2,3,4], Tiannan Guo[2,3,4], Hongkun Wu[7,8*], Hao Zhang[1*], Yaoting Sun[2,3,4*]

[1]Department of Thyroid Surgery, the First Hospital of China Medical University, No. 155 Nanjing North Street, Shenyang 110001, China;
[2]Westlake Center for Intelligent Proteomics, Westlake Laboratory of Life Sciences and Biomedicine, No. 18 Shilongshan Road, Hangzhou 310024, China;
[3]School of Medicine, School of Life Sciences, Westlake University, No. 18 Shilongshan Road, Hangzhou 310024, China;
[4]Research Center for Industries of the Future, Westlake University, No. 600 Dunyu Road, Hangzhou 310030, China;
[5]College of Pharmaceutical Sciences, Zhejiang University, No. 866 Yuhangtang Road, Hangzhou 310058, China;
[6]Westlake Omics (Hangzhou) Biotechnology Co., Ltd., No. 1 Yunmeng Road, Hangzhou 310024, China;
[7]Department of Hepatobiliary and Pancreatic Surgery, the First Affiliated Hospital, Zhejiang University School of Medicine, No. 79 Qingchun Road, Hangzhou 310003, China;
[8]Zhejiang Provincial Key Laboratory of Pancreatic Disease, No. 79 Qingchun Road, Hangzhou 310003, China.

[#]These authors contributed equally: Zhihong Wang, He Wang, Yan Zhou;
[*]These authors jointly supervised this work: Yaoting Sun (sunyaoting@westlake.edu.cn); Hao Zhang (haozhang@cmu.edu.cn); Hongkun Wu (wuhongkun@zju.edu.cn).

Supplementary Table 1. Enriched keywords of 27 dysregulated proteins in PM

Term description	Observed symbol count	Background symbol count	Strength#	False discovery rate*	Proteins
Immunity	7	537	0.92	0.0058	OAS3, HLA-DOA, GZMM, PRG2, OAS2, HLA-DPA1, PRKCB
MHC-II	2	12	2.02	0.0481	HLA-DOA, HLA-DPA1

Note:

#Strength indicates how large the enrichment effect is and is calculated by $\log_{10}$(observed/expected).

*False discovery rate represents the *P* values corrected for multiple testing by the Benjamini–Hochberg procedure.

Supplementary Table 2. Final hyperparameters setting and feature set

Model and mlr3learner used	Final hyperparameters setting	Feature set
CliCox<br>lrn ("surv.coxph")	NA	11 clinical features
CliRsf<br>lrn ("surv.rfsrc")	nodesize = 8<br>ntree = 1000<br>mtry = 3	11 clinical features
ProtCox<br>lrn ("surv.glmnet")	lambda = 0.1116327<br>alpha = 1	"P10645" "P17931" "P22897" "P23378" "P27635" "Q06033" "Q6PHW0"
ProtRsf<br>lrn ("surv.rfsrc")	nodesize = 6<br>ntree=1000<br>mtry = 4	"Q8TBF5" "P10645" "P12111" "Q96RP7" "O00584" "L0R819" "P17931" "Q99972" "P13612" "Q9Y4Z0" "Q4G0X9" "Q96AN5" "Q9NQ79" "Q86Y22" "Q96F24" "Q9BXJ5" "Q96JY6" "Q08495" "P23378"
CliProtRsf<br>lrn ("surv.rfsrc")	nodesize = 7<br>ntree=1000<br>mtry = 5	"Q8TBF5" "P12111" "P10645" "O00584" "Q96RP7" "L0R819" "P17931" "Q99972" "P13612" "Q9Y4Z0" "O75478" "Q4G0X9" "Q86Y22" "Q8N4C6" "Q9Y6R1" "Q96AN5" "Q9BXJ5" "Q96F24" "Q9NQ79" "Q08495" "P23378" "Q6ZU35"

Supplementary Table 3. The 19 selected protein features

UniProt ID	Gene name	Protein name	Thyroid cancer related	Thyroid function related
P10654	CHGA	Chromogranin-A (CGA, PHE5, PHES)	Yes	-
Q86Y22	COL23A1	Collagen alpha-1(XXIII) chain	Yes	-
P12111	COL6A3	Collagen alpha-3(VI) chain	Yes	-
P13612	ITGA4	Integrin alpha-4	Yes	-
P17931	LGALS3	Galectin-3	Yes	-
Q99972	MYOC	Myocilin	-	Yes
Q08495	DMTN	Dematin	-	-
Q8TBF5	PIGX	Phosphatidylinositol glycan anchor biosynthesis class X	-	-
O00584	RNASET2	Ribonuclease T2	-	-
Q96RP7	GAL3ST4	Galactose-3-O-sulfotransfaerase 4	-	-
Q4G0X9	CCDC40	Coiled-coil domain-containing protein 40	-	-
Q96JY6	PDLIM2	PDZ and LIM domain protein 2	-	-
P23378	GLDC	glycine decarboxylase	-	-
Q9BXJ5	C1QTNF2	Complement C1q tumor necrosis factor-related protein 2	-	-
Q96F24	NRBF2	Nuclear receptor-binding factor 2	-	-
Q9Y4Z0	LSM4	U6 snRNA-associated Sm-like protein LSm4	-	-
L0R819	ASDURF	ASNSD1 upstream open reading frame protein	-	-
Q9NQ79	CRTAC1	Cartilage acidic protein 1	-	-
Q96AN5	TMEM 143	Transmembrane protein 143	-	-

Supplementary Table 4. Predicted transcription regulators of the 19 protein features

Molecule Type	Upstream Regulator	*P* value of overlap*	Target Molecules in Dataset
transcription regulator	SREBF1	0.000977	GLDC, LGALS3, RNASET2
transcription regulator	ERCC6	0.00208	LGALS3
transcription regulator	CBFA2T2	0.00277	CHGA
transcription regulator	EOMES	0.00285	COL6A3, ITGA4

Note:

**P* values are calculated by Fisher's exact test.

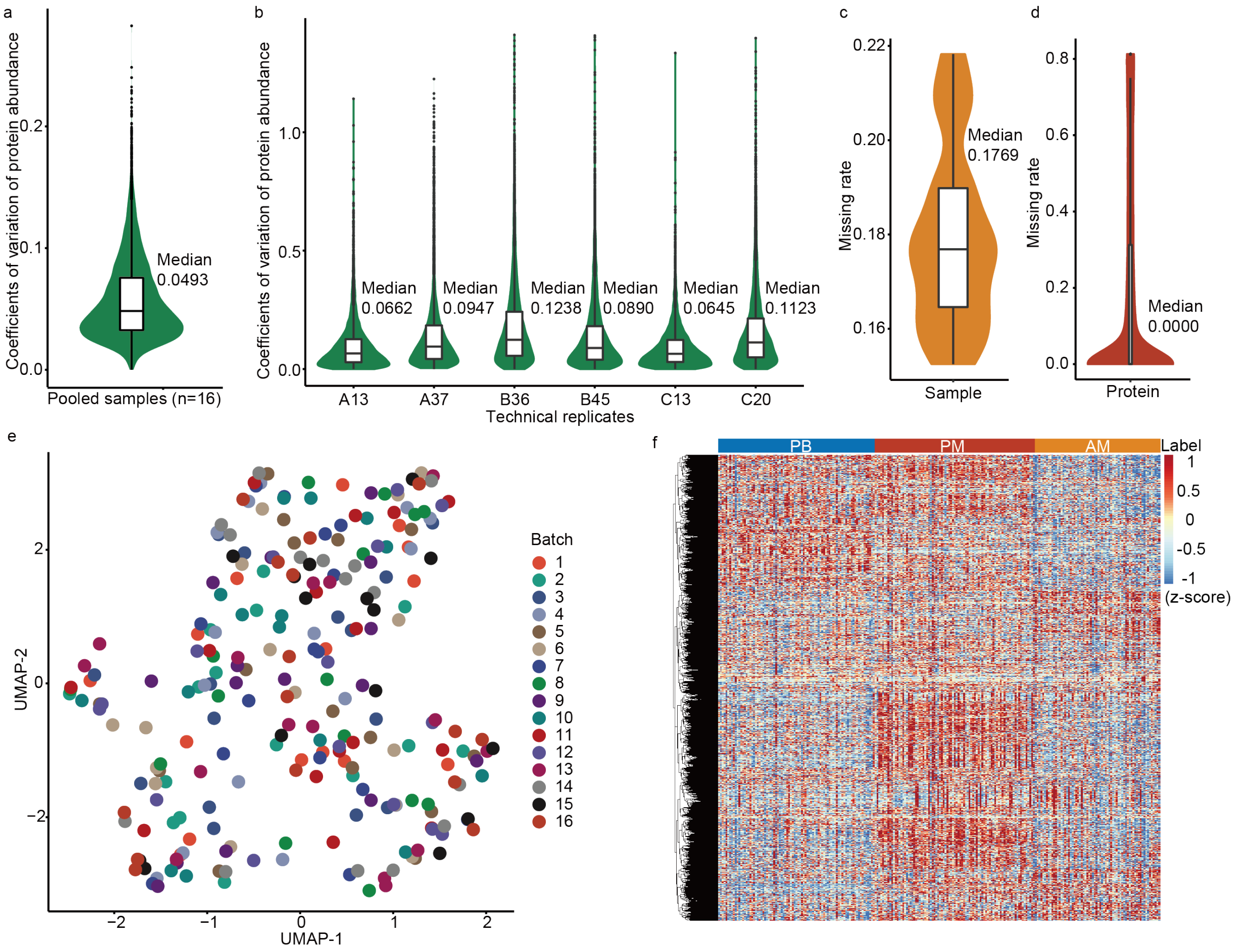

Supplementary Figure 1. Data quality control and preprocessing. (a) CVs of the protein abundances across pooled samples. (b) CVs of the protein abundances across technical replicates. (c-d) Violin plots showing the missing value rates of (c) samples and (d) proteins. (e) UMAP visualization of 240 thyroid tissue samples grouped in 16 batches. (f) Heatmap of 9154 proteins from 240 thyroid tissue samples.

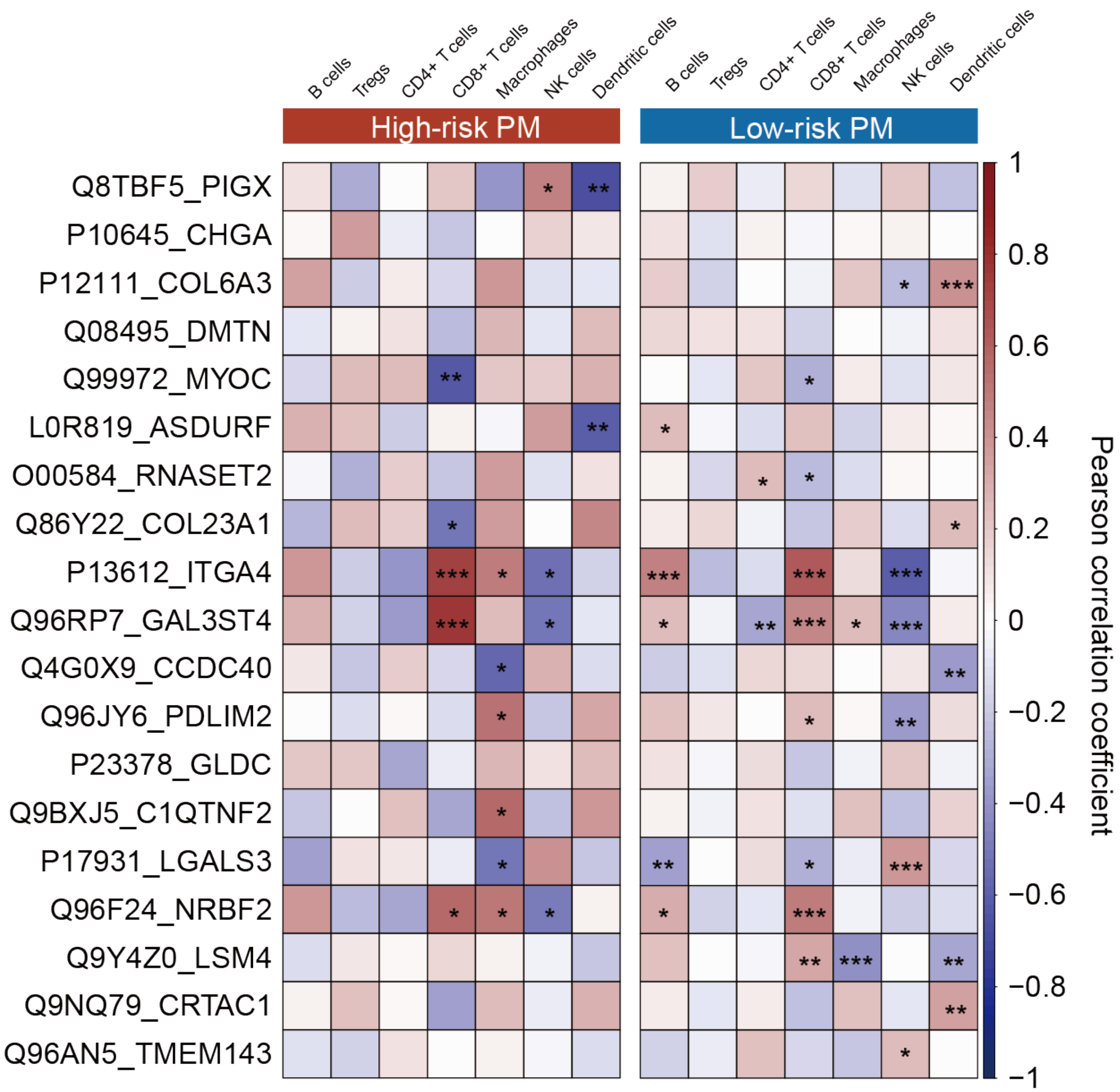

Supplementary Figure 2. Pearson correlations of immune cell fractions and the 19 proteins from the ProtRsf model. The plots showing the Pearson correlation coefficient of high- and low-risk of disease recurrence groups which were predicted by our ProtRsf model (*$P < 0.05$, **$P < 0.01$ and ***$P < 0.001$).

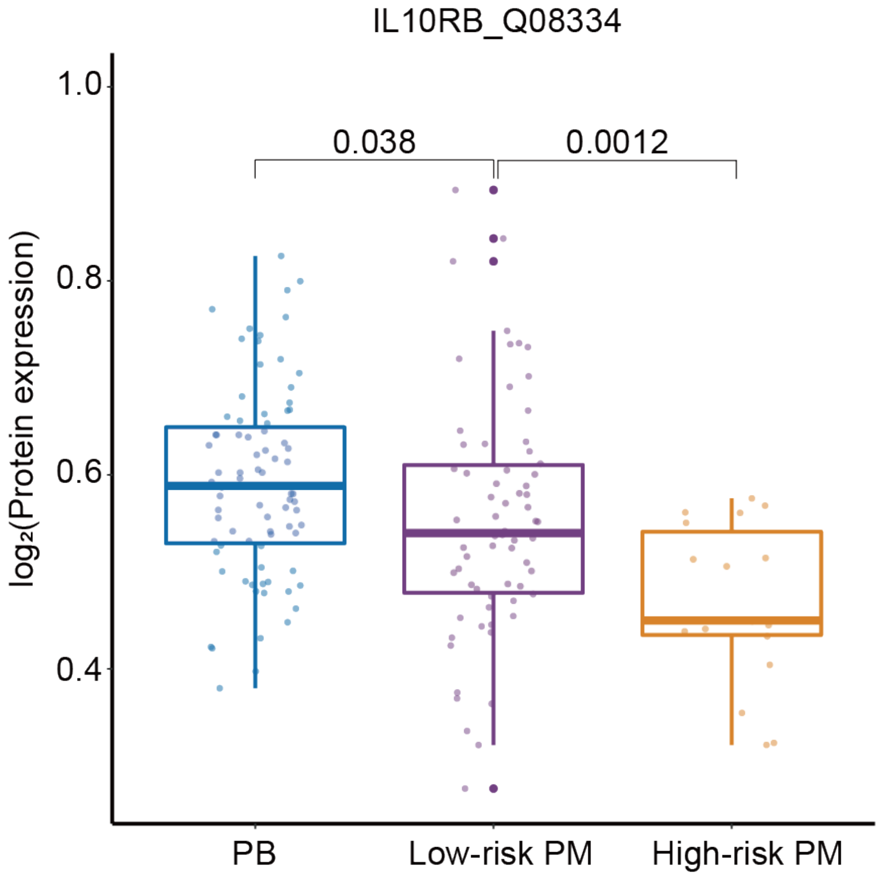

Supplementary Figure 3. The abundance of immune checkpoint interleukin 10 receptor B (IL10RB) in pediatric benign (PB), low-risk and high-risk pediatric malignant (PM) samples. The low- and high-risk groups were predicted by the ProtRsf model. Boxes are first and third quartile, the center line is median, whiskers are ± 1.5 interquartile range, and dots are indicated individual data points. Abundance outliers (defined as 1.5 times interquartile range) and missing values are not shown in the boxplot. Biologically independent samples shown in boxplot: PB, N=83; Low-risk PM, N=67; High-risk PM, N=18. *P* values are derived from two-sided Welch's *t*-test.
