# Supplementary material for: An individualized protein-based prognostic model to stratify pediatric patients with papillary thyroid carcinoma
Source: Nat Commun. 2024 Apr 26;15:3560. doi: 10.1038/s41467-024-47926-w (PMC11053152; doi:10.1038/s41467-024-47926-w)
Supplement: Supplementary file 3 — Description of Additional Supplementary Files [file 41467_2024_47926_MOESM3_ESM.docx]

Description of Additional Supplementary Files

File Name: Supplementary Data 1

Description: Clinical characteristics of the pediatric cohort

File Name: Supplementary Data 2

Description: Protein matrix

File Name: Supplementary Data 3

Description: Differentially expressed proteins in PM vs. PB and PM vs. AM

File Name: Supplementary Data 4

Description: Enriched pathways and GO biological functions for DEP
