# Supplementary material for: An individualized protein-based prognostic model to stratify pediatric patients with papillary thyroid carcinoma
Source: Nat Commun. 2024 Apr 26;15:3560. doi: 10.1038/s41467-024-47926-w (PMC11053152; doi:10.1038/s41467-024-47926-w)
Supplement: Supplementary file 8 — Reporting Summary [file 41467_2024_47926_MOESM8_ESM.pdf]

nature portfolio

Corresponding author(s): Yaoting Sun

Last updated by author(s): Mar 5, 2024

Reporting Summary

Nature Portfolio wishes to improve the reproducibility of the work that we publish. This form provides structure for consistency and transparency in reporting. For further information on Nature Portfolio policies, see our Editorial Policies and the Editorial Policy Checklist.

Statistics

For all statistical analyses, confirm that the following items are present in the figure legend, table legend, main text, or Methods section.

n/a	Confirmed	
☐	☒	The exact sample size (*n*) for each experimental group/condition, given as a discrete number and unit of measurement
☐	☒	A statement on whether measurements were taken from distinct samples or whether the same sample was measured repeatedly
☐	☒	The statistical test(s) used AND whether they are one- or two-sided<br>*Only common tests should be described solely by name; describe more complex techniques in the Methods section.*
☐	☒	A description of all covariates tested
☐	☒	A description of any assumptions or corrections, such as tests of normality and adjustment for multiple comparisons
☐	☒	A full description of the statistical parameters including central tendency (e.g. means) or other basic estimates (e.g. regression coefficient) AND variation (e.g. standard deviation) or associated estimates of uncertainty (e.g. confidence intervals)
☐	☒	For null hypothesis testing, the test statistic (e.g. *F*, *t*, *r*) with confidence intervals, effect sizes, degrees of freedom and *P* value noted<br>*Give P values as exact values whenever suitable.*
☒	☐	For Bayesian analysis, information on the choice of priors and Markov chain Monte Carlo settings
☒	☐	For hierarchical and complex designs, identification of the appropriate level for tests and full reporting of outcomes
☒	☐	Estimates of effect sizes (e.g. Cohen's *d*, Pearson's *r*), indicating how they were calculated

Our web collection on statistics for biologists contains articles on many of the points above.

Software and code

Policy information about availability of computer code

Data collection	Sample were analyzed using liquid chromatography-mass spectrometry (nanoflow DIONEX UltiMate 3000 RSLCnano System and Orbitrap Exploris 480 with FAIMS Pro™, Thermo Fisher Scientific, USA).
Data analysis	Proteomic raw files were searched using Proteome Discoverer (v2.4.1.15) against a FASTA file containing 20,368 entries (human Swiss-Prot database). CIBERSORTx15 (https://cibersortx.stanford.edu/) was utilized to profile the proportions of 7 immune cell types in our proteomic data. Statistical analysis was conducted using R (v4.1.1) and SPSS (v 23.0). The R package NAguideR was used for missing value imputation, and the impseqrob method (for robust sequential imputation) was used. The batch effects correction of the resulting protein matrix was performed using Combat, an empirical Bayes framework from the R package sva. Approximation and Projection (UMAP) visualization was performed using the R package UMAP. The heatmap was generated using the R package pheatmap. Machine learning models were built through R package mlr3. GO enrichment analysis was conducted by enrichGO function in R Package clusterProfiler (v4.0.5) using database org.Hs.eg.db (v3.13.0, stored in R package org.Hs.eg.db). Pathways and networks were analyzed using the Ingenuity Pathway Analysis (IPA) and visualized with Cytoscape (v3.8.2).

For manuscripts utilizing custom algorithms or software that are central to the research but not yet described in published literature, software must be made available to editors and reviewers. We strongly encourage code deposition in a community repository (e.g. GitHub). See the Nature Portfolio guidelines for submitting code & software for further information.

Data

Policy information about availability of data

All manuscripts must include a data availability statement. This statement should provide the following information, where applicable:

- Accession codes, unique identifiers, or web links for publicly available datasets
- A description of any restrictions on data availability
- For clinical datasets or third party data, please ensure that the statement adheres to our policy

The mass spectrometry proteomic raw data generated in this study have been deposited to the ProteomeXchange Consortium via the iProX partner repository under accession identifier IPX0006407000 (subproject ID: IPX0006407001) [https://www.iprox.cn/page/project.html?id=IPX0006407000 or https://proteomecentral.proteomexchange.org/cgi/GetDataset?ID=PXD050347].

Research involving human participants, their data, or biological material

Policy information about studies with human participants or human data. See also policy information about sex, gender (identity/presentation), and sexual orientation and race, ethnicity and racism.

Reporting on sex and gender	In this study, the findings apply to both sexes. We enrolled 234 patients with pediatric thyroid carcinoma (N=85) , pediatric benign nodules (N=83) and adult thyroid papillary thyroid carcinoma (N=66). This cohort included 55 males and 179 females.
Reporting on race, ethnicity, or other socially relevant groupings	All samples analyzed were from Asian ethnic groups. Ethnicity was not a relevant categorical variable for this study.
Population characteristics	The overall study design was demonstrated in Figure 1A. We enrolled 85 PPTC patients (PM), and collected their clinicopathological features (Figure 1B and Supplementary Table 1). This group included 23 males and 62 females, with a male-to-female ratio of 1:2.5 and an average age of 15.7 ± 2.4 years (ranging from 8.0 to 18.0 years). All 85 patients were admitted to the hospital with a mass in the neck, and their average tumor size was 2.4 ± 1.3 cm (ranging from 0.3 to 7.0 cm). Besides the neck mass, one patient lamented hoarseness and another neck pain. Additionally, preoperative pulmonary computed tomography (CT) showed that one patient had multiple metastases in the lung. All patients were surgically treated; 47 (55.29%) underwent lobectomy, and 38 (44.71%) had a total thyroidectomy. Prophylactic central neck lymph node dissections were performed in all patients, while modified lateral neck dissections were performed in 43 patients. We recorded 16 cases (18.82%) with multifocal disease, 69 (81.18%) with lymph node metastases and 43 cases (43/85, 50.59%) of lateral cervical lymph node metastases.
Recruitment	In this retrospective study, we evaluated pediatric patients (≤18 years) with thyroid nodules, including 85 pediatric malignant (PM) and 83 pediatric benign (PB) thyroid nodules, who underwent surgery in the First Hospital of China Medical University between November 2007 and April 2021.<br>There was no selection bias.
Ethics oversight	This study protocol and waiver of informed consent was approved by the Ethics Committee of the First Hospital of China Medical University with the study number 2021-287-2.

Note that full information on the approval of the study protocol must also be provided in the manuscript.

Field-specific reporting

Please select the one below that is the best fit for your research. If you are not sure, read the appropriate sections before making your selection.

☒ Life sciences ☐ Behavioural & social sciences ☐ Ecological, evolutionary & environmental sciences

For a reference copy of the document with all sections, see nature.com/documents/nr-reporting-summary-flat.pdf

Life sciences study design

All studies must disclose on these points even when the disclosure is negative.

Sample size	We collected 240 thyroid nodules (87 PM, 85 PB, 68 AM) FFPE slides (10 μm thick) from 234 patients (85 PM, 83 PB, 66 AM). The sample size was chosen based on the data that were available when the study was launched since no sample size estimates had been made.
Data exclusions	The exclusion criteria for PM were the following: (a) with a history of radiation exposure or family history, (b) with poorly differentiated PTC, (c) loss of follow-up or incomplete clinical data, and (d) non-primary operation. We excluded uncertain malignant potential nodules for the PB group.
Replication	Two samples from each group were randomly selected as technical replicates. All attempts at replication were successful.
Randomization	The 240 collected tissues were randomized into 16 batches with 15 tissue samples each, and one pooled sample was used as a linker for the batches.

Blinding

To ensure that the samples collected are the correct targets, there were no blinding during the sample collection. The investigators who prepared the samples were blinded to the clinical information. The investigators were blinded to group allocation during model test analysis.

Reporting for specific materials, systems and methods

We require information from authors about some types of materials, experimental systems and methods used in many studies. Here, indicate whether each material, system or method listed is relevant to your study. If you are not sure if a list item applies to your research, read the appropriate section before selecting a response.

Materials & experimental systems

n/a	Involved in the study
☐	☒ Antibodies
☒	☐ Eukaryotic cell lines
☒	☐ Palaeontology and archaeology
☒	☐ Animals and other organisms
☒	☐ Clinical data
☒	☐ Dual use research of concern
☒	☐ Plants

Methods

n/a	Involved in the study
☒	☐ ChIP-seq
☒	☐ Flow cytometry
☒	☐ MRI-based neuroimaging

Antibodies

Antibodies used

CD3 (cat# ab135372, dilution 1:500, Abcam), CD4 (cat# ab288724, dilution 1:1000, Abcam), and CD8 (cat# ab17147, dilution 1:500, Abcam)

Validation

All antibodies used in this study were obtained from the indicated commercial vendors and validated by the respective manufacturer as described in their website.
Anti-CD3 (cat# ab135372, dilution 1:500, Abcam) validated for Immunofluorescent by manufacturer: [https://www.abcam.cn/products/primary-antibodies/cd3-antibody-sp162-ab135372.html],
Anti-CD4 (cat# ab288724, dilution 1:1000, Abcam) validated for Immunofluorescent by manufacturer: [https://www.abcam.cn/products/primary-antibodies/cd4-antibody-rm1013-ab288724.html],
Anti-CD8 (cat# ab17147, dilution 1:500, Abcam) validated for Immunofluorescent by manufacturer: [https://www.abcam.cn/products/primary-antibodies/cd8-alpha-antibody-c8144b-ab17147.html].
